# Direct targeting of DOCK4 by miRNA-181d in oxygen-glucose deprivation/reoxygenation-mediated neuronal injury

**DOI:** 10.1186/s12944-023-01794-3

**Published:** 2023-03-07

**Authors:** Shengnan Li, Shaofeng Chen, Yajun Wang, Xingjuan Hu, Ying Wang, Zhaochun Wu, Shaoting Huang, Jiawen He, Fu Deng, Bin Zhao, Guoda Ma, You Li

**Affiliations:** 1 Guangdong Key Laboratory of Age-Related Cardiac and Cerebral Diseases, Affiliated Hospital of Guangdong Medical University, Zhanjiang, 524001 China; 2 Department of Neurology, Affiliated Hospital of Guangdong Medical University, Zhanjiang, 524001 China; 3 Institute of Neurology, Affiliated Hospital of Guangdong Medical University, Zhanjiang, 524001 China; 4 Maternal and Children’s Health Research Institute, Shunde Maternal and Children’s Hospital, Guangdong Medical University, Shunde, 528300 China

**Keywords:** miRNA-181d, DOCK4, Ischemic stroke, OGD/R, Polymorphism

## Abstract

The miRNA-181 (miR-181) family regulates neuronal persistence during cerebral ischemia/reperfusion injury (CI/RI). Since the effect of miR-181d on CI/RI has never been studied, the current work sought to determine the involvement of miR-181d in neuronal apoptosis after brain I/R injury. To replicate in vivo and in vitro CI/RI, a transient middle cerebral artery occlusion (tMCAO) model in rats and an oxygen-glucose deficiency/reoxygenation (OGD/R) model in neuro 2A cells were developed. In both in vivo and in vitro stroke models, the expression of miR-181d was considerably higher. miR-181d suppression reduced apoptosis and oxidative stress in OGD/R-treated neuroblastoma cells, but miR-181d overexpression increased both. Furthermore, it was observed that miR-181d has a direct target in dedicator of cytokinesis 4 (DOCK4). The overexpression of DOCK4 partially overcame cell apoptosis and oxidative stress induced by miR-181d upregulation and OGD/R injury. Furthermore, the DOCK4 rs2074130 mutation was related to lower DOCK4 levels in ischemic stroke (IS) peripheral blood and higher susceptibility to IS. These findings suggest that downregulating miR-181d protects neurons from ischemic damage by targeting DOCK4, implying that the miR-181d/DOCK4 axis might be a novel therapeutic target for IS.

**Graphical Abstract:**

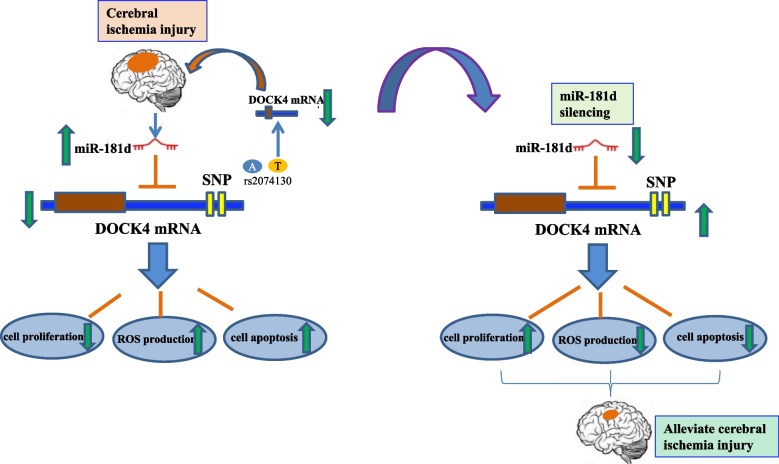

**Supplementary Information:**

The online version contains supplementary material available at 10.1186/s12944-023-01794-3.

## Introduction

Ischemic stroke (IS) is one of the primary severe cerebrovascular disorders worldwide, leading to death and long-term disability [1]. Currently, intravenous injection of recombinant tissue plasminogen activator (tPA) persists as the primary means of treating IS. However, the limited time window for thrombolysis dramatically limits its application, and the risk of intracranial hemorrhage may outweigh the benefits [2]. After thrombolysis, blood arteries recanalize, resulting in reperfusion damage and possibly poor prognosis. Studies have demonstrated that stroke causes neuronal cell death, including necrosis, apoptosis, pyroptosis, ferroptosis, and autophagy [3]. Nevertheless, the exact mechanism of stroke-induced cell death remains unclear. Therefore, a detailed understanding of the molecular pathway of neural cell death will undoubtedly benefit the development of neuroprotective drugs for stroke.

MicroRNAs (miRNAs) are short (approximately 17-25 nt) noncoding RNAs that can control gene expression by suppressing mRNA translation/bringing degradation by the complementary pairing of oligonucleotides with target messenger RNA [4]. miRNA dysregulation has been implicated in a variety of neurological disorders, such as Alzheimer’s [5], Parkinson’s [6], and IS [7]. The miR-181 family emerges as essential regulators in vascular inflammation by regulating targets related to endothelial cell activation and immune cell homeostasis [8–10]. Previous studies have shown the potential of miR-181d in promoting neurite growth of PC-12 cells through the PI3K/Akt signaling mechanism, signifying it as a potential therapeutic target of spinal cord injury [11]. Wang et al. reported that miR-181d regulates axon elongation by targeting Map1b and Calm1 [12]. The role of miR-181d in CI/RI remains largely unknown.

Cytokinesis 4 (DOCK4) dedicators involved in dendritic spine development have been identified as risk genes for autism spectrum disorder (ASD), dyslexia, and schizophrenia [13]. Moreover, studies have demonstrated that the rs2074130 and rs2217262 polymorphisms of DOCK4 are associated with the risk of autism [14] and schizophrenia [15], respectively. Xiao et al. suggested that DOCK4 may exert a role in neurite differentiation and dendrite arborization [16]. Huang et al. discovered that DOCK4 mutants were defective in their ability to promote neurite outgrowth and dendritic spine formation [15]. In addition to neurite formation and development, DOCK4 also regulates vascular endothelial cell function. Several studies have reported DOCK4’s involvement in promoting vascular smooth muscle cell (VSMC) migration [17], vessel sprouting, and tubule remodeling [18]. Additionally, a recent study reported that DOCK4 promotes the internalization of scavenger receptor class B type 1 and LDL transport by activating the RHO GTPase RAC1, which may promote atherosclerosis in endothelial cells [19]. Despite these advances, the role of DOCK4 in cerebral I/R is still unclear.

Both in vivo (MCAO) and in vitro (OGD/R) models of CI/RI were used to investigate the kinetics of miR-181d expression in this situation. Gain and loss of functional tactics were applied to clarify the regulatory mechanisms of miR-181d in the case of OGD/R-induced cellular damage. The role of the miR-181d/DOCK4 axis in ischemic damage was also examined. To determine whether DOCK4 variants are associated with genetic susceptibility to IS, additional analyses of the rs2074130 and rs2217262 DOCK4 variants were also conducted. These results suggest that the miR-181d/DOCK4 axis and DOCK4 variants may play a pathogenic role in IS.

## Materials and methods

### MCAO and animal model

All of the animal experiments mentioned in this article received approval from the Guangdong Medical University Ethics Committee for Animals following the NIH Care Guidelines and usage of experimental animals. Male Sprague Dawley (SD) mice (weight range from 280 to 320 g) were anesthetized with an intraperitoneal dose of ketamine (75 mg/kg) and 3% isoflurane. The transient MCAO model experiment was carried out as reported earlier [20]. After anesthesia, the remaining internal and external carotid arteries were surgically removed via an endoscopic midline incision. After 24 h, the middle cerebral artery was sealed with a round nylon (4-0 suture) tip, and the junction was detached to allow blood flow restoration. The control rats (sham group) underwent identical operations, but the middle cerebral artery was unblocked. The experimental animals that underwent surgery were treated with heating pads to keep their body temperatures at 37 ± 0.5 °C. The rats were sacrificed 24 h after MCAO modeling. Subsequently, infarcted brain tissue was isolated and cut into 3-mm-thick sections for staining with 2,3,5-triphenyl tetrazolium chloride (TTC) (Sigma, MO, USA).

### Cell cultivation and OGD/R modeling

An OGD/R cell model was prepared as reported previously [21]. In MEM (Gibco, CA, USA) supplemented with FBS (10%) and 1% streptomycin or penicillin (Gibco, CA, USA) in 5% carbon dioxide and incubated at 37 °C, murine N2a neuroblastoma cells (ATCC, Shanghai Cell Bank) were cultured. After replacing the culture medium with glucose-free Dulbecco’s Modified Eagle Medium (DMEM) from Gibco (CA, USA), OGD was induced. N2a cells were cultured under 1% oxygen, 94% nitrogen, and 5% carbon dioxide for 4 h at 37 °C. Afterward, the cells were transferred back to normoxic circumstances in standard media for 3, 6, 12, and 24 h to facilitate reoxygenation.

### Cell transfection

The miR-181d mimics, miR-mimic control (mimics NC), miR-181d inhibitor, and miR-inhibitor control (inhibitor NC) were purchased from GenePharm (Shanghai, China). Full-length DOCK4 fragments were cloned into the pcDNA3.0 plasmid purchased from Santa Cruz Biotechnology (Santa Cruz, CA, USA). Cell transfection was conducted via Lipofectamine™ 2000 (Invitrogen) following the manufacturer’s instructions.

### Quantitative real-time polymerase chain reaction (RT–qPCR) assay

TRIzol (Qiagen, CA, USA) was employed to obtain total RNA from brain tissue and cell samples. Subsequently, the SuperScript II First-Strand cDNA Synthesis Kit (Thermo Fisher Scientific, CA, USA) was implemented to synthesize the corresponding cDNA. RT–qPCR was performed using the SYBR Premix ExTag TMII kit (Takara, Japan) on an ABI Prism 7900 instrument (Applied Biosystems, CA, USA). Relative gene expression was evaluated using 2^-ΔΔCt^ with GAPDH as a normalized control. The primer sequences utilized in this study are listed below:

- MiR-181d-F: 5′- GGGAACATTCATTGTTGTCG − 3′;
- MiR-181d-R: 5′-CAGTGCGTGTCGTGGAGT-3′;
- DOCK4-F: 5′- GATAGGAGAGGTGGATGGCAAG -3′;
- DOCK4-R: 5′- CGCCTTGAGATGCAGATCGTAG-3′;
- GAPDH-F: 5′- AGGTCAATGAAGGGGTCGTT-3′;
- GAPDH-R: 5′- AAATGGTGAAGGTCGGTGTG-3′

### Cell viability assay

A 3-(4,5-dimethylthiazol-2-yl)-2,5-diphenyltetrazolium bromide (MTT) test was performed to assess cellular viability. In brief, N2a cells were subjected to 96-well culture plates. They were cultured overnight, subsequently transfected with suitable miRNA, inhibitor, and/or siRNA constructs and incubated for another 24 h before OGD/R treatment. Fresh media was replaced, and MTT reagents (Sigma, CA, USA) were added and incubated at 37 °C for an extra 4 h. Optical density values were assessed at 490 nm. They were used to compute the viability of the cell.

### Lactate dehydrogenase (LDH) assay

Following the specified instructions, quantification of LDH proclamation was performed using a CytoTox 96® Non-Radioactive Cytotoxicity Assay kit (Promega Corporation, USA). A lactate dehydrogenase reaction substrate was used in place of the medium for an hour at a temperature of 37 °C, and cells were treated the same way as in MTT experiments. A microplate reader (*BioTek* Instruments, Inc. USA) was used to measure absorbance/optical density at 490 nm in a mixture of supernatant and LDH detection reagents for 30 min.

### Dichlorofluorescein diacetate (DCFH-DA) assay

The DCFH-DA assay was carried out precisely as reported previously [22]. N2a cells were treated for approximately 20 min to recognize ROS at 37 °C with the dichlorofluorescein diacetate probe. Flow cytometry (FACSCanto II, BD, USA) was used to remove the supernatant and collect cells for DCFH-DA staining.

### Apoptosis assay

OGD/R-induced cellular apoptosis was determined by the Annexin V-FITC/PI test. In brief, N2a cells were transfected with suitable miRNA mimics, inhibitors, and/or siRNA constructs for approximately 24 h before OGD/R therapy. Next, resuspended cells were collected and incubated with binding buffer (supplemented with 5 μL of annexin V-FITC and PI) for approximately 15 min at room temperature. A FACSCanto II flow cytometer (BD, USA) was utilized to identify apoptotic cells.

### Dual-luciferase reporter assay

The wild-type DOCK4 3′-UTR contains the miR-181d binding site seed sequence or a mutant (MUT); isoform cloning was performed in the dual-luciferase vector pmirGLO. N2a cell plating was performed in plates containing 24 wells, cotransfected with the WT or mutant constructs of DOCK4 and with miR-181d mimics or equivalent controls using Lipofectamine 2000 (Invitrogen, CA, USA). At 48 h posttransfection, relative luciferase activity was evaluated via a Dual-Luciferase Reporter Assay Kit (Promega, USA).

### Western blotting

As previously stated, RIPA buffer containing proteinase inhibitors was utilized to extract total protein from N2a cells or rat brain cortex [23]. A BCA assay was applied to measure the lysed protein concentrations. Then, proteins from individual samples prepared above were separated by 10% SDS–PAGE and transferred to PVDF membranes overnight at 4 °C. Then, the membrane was probed with appropriate primary antibodies, namely, DOCK4 (CST; 1:500), Bcl2, Bax, caspase3, and GAPDH (Abcam; 1:1000). Blots were subsequently probed using secondary HRP-conjugated goat anti-rabbit IgG antibodies (Abcam; 1:2000) for approximately 90 min at ambient temperature. An improved chemiluminescence kit (Pierce, Thermo Fisher Scientific, Inc.) was employed to detect proteins.

### Patient characteristics

The Ethics Committee of Guangdong Medical College’s Affiliated Hospital approved the study. Before the investigation, a letter of information agreement was obtained from each member enlisting. Between 2015 and 2019, 1045 age-matched healthy controls and 1086 IS patients were tested at Guangdong Medical University’s Associated Hospital. Clinical signs and symptoms, computed tomography (CT) scans, and magnetic resonance imaging (MRI) tests were used to examine patients with IS independence. Exceptions were made for patients with a history of CTI (cerebral, transient ischemia), subarachnoid hemorrhage, and autoimmune illnesses (such as malignant tumors, chronic infections, hematological, systemic inflammatory, or coronary artery disease). The controls in this study did not have tumors with malignant status, autoimmunity, chronic inflammation, or an IS history. Prior measurements defined high blood pressure, smoking, and diabetes [24].

### Genotyping of DOCK4 variants

According to past studies, two DOCK4 variants (rs2217262 and rs2074130) were selected [14, 25]. Peripheral blood mononuclear cell samples were used to isolate genomic DNA through a DNA purification kit (Sangon Biotech, China). The rs2217262 and rs2074130 genotyping were achieved using an improved multiplex, ligase-detection reaction technique utilizing the sequences of the primers as follows:

- rs2217262-F: 5′-GTTTCCATATTCTACTGTGT TGTTCC-3′,
- rs2217262-R: 5′-TAGACAGAGAGGCGCTATGTACC-3′, and
- extension primer: 5′-TTCCGCGTTCGGACTGATATGCCTGGTACACTGCCAGTCG-3′;
- rs2074130-F: 5′-TTCTCCTTAATCCCTTTCCTTTTTC-3′,
- rs2074130-R: 5′-TCCTTCCTACATCCATGTTCATTTC-3′, and
- extension primer: 5′-TCTCTCGGGTCAATTCGTCCTTTGGTGACTCTTGCACA GAGTCG-3′.

### Statistical analyses

GraphPad Prism 5.0 (GraphPad, CA, USA) was used to examine the statistical data, presented as the means and the standard deviation (±SD) from triplicate experiments. Data comparisons among groups were made through one-way ANOVAs or Student’s t tests. The categorical variables and Hardy-Weinberg equilibrium (HWE) were related using the *X*^*2*^ test for the gene polymorphism analysis. Student’s t tests were used to analyze consecutive data. If the information was not distributed as expected, the Mann–Whitney U test was applied. The additive dominant and recessive models link genotype distribution between patients and controls. Haplotype investigation was performed with Haplo-view v4.2 software. The connection between definite variants and IS risk was assessed through OR (odds ratios) with a 95% CI (confidence interval) after modifying several criteria, i.e., hyperlipidemia, diabetes mellitus, smoking, high blood pressure, sex, and age. For several differences considering control type-1 error, Bonferroni correction was applied in the statistical analysis. *P* < 0.05 was set as the significance threshold.

## Results

### The expression of miR-181d and DOCK4 in stroke models

**Fig. 1:**
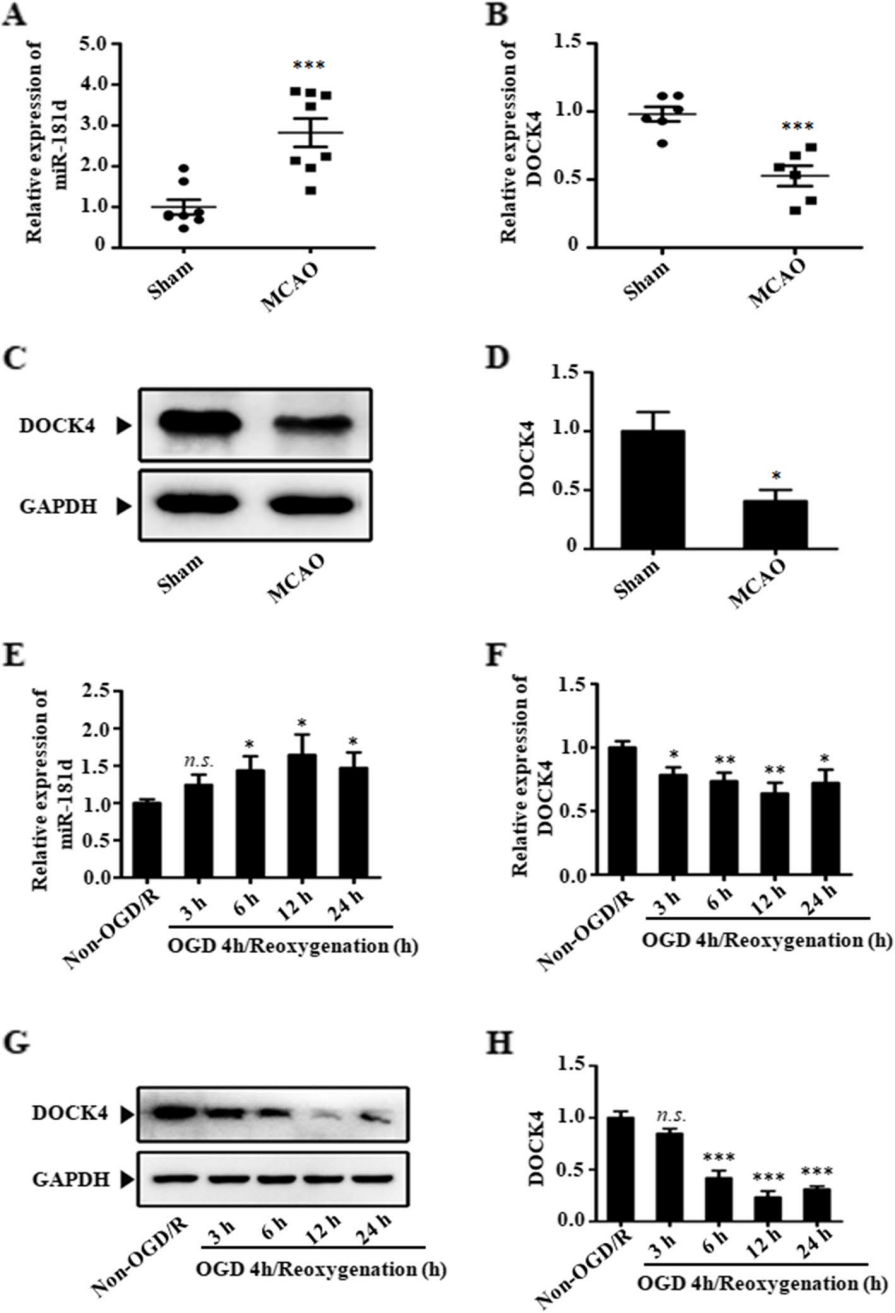
The expression of miR-181d and DOCK4 in in vivo and in vitro models of IS. **A**&**B** Rats were subjected to sham and MCAO for 2 h, followed by 24 h of reperfusion. Then, brain tissues were harvested and detected with qRT–PCR. The whiskers indicate error bars. **C**&**D** The expression of DOCK4 in brain tissues was quantified by western blotting. **E**&**F** N2a cells were exposed to OGD for 4 h and reoxygenation for 3, 6, 12, and 24 h. The expression of miR-181d and DOCK4 was determined by qRT–PCR. **G**&**H** The expression of DOCK4 in N2a cells exposed to OGD for 4 h and reoxygenation for 3, 6, 12, and 24 h was quantified by western blotting. Cells cultured in normal medium and normoxic conditions served as a control (n.s., not significant, *P < 0.05, **P < 0.01, ***P < 0.001)

The present study employed a mouse model to determine how miR-181d expression affects ischemia injury. Animals were subjected to a 2-h MCAO method and a 24-h reperfusion duration. Furthermore, exposing N2a cells to OGD and reperfusion several times was employed to develop an in vitro model of IS. Then, the miR-181d levels in N2a cells under OGD/R conditions and in infarcted cortical tissues were investigated. MiR-181d levels in the cerebral infarction core of MCAO animals were substantially higher than those in sham controls (Fig. 1A). Likewise, there was a considerable increase in miR-181d levels in OGD/R-treated N2a cells, with expression increasing as the reoxygenation time was prolonged from 3 to 12 h but with downregulation after 24 h of reoxygenation (Fig. 1E).

Because bioinformatics research revealed that DOCK4 might be a direct target of miR-181d, DOCK4 expression alterations in stroke models were also examined. qPCR and western blotting analysis showed that DOCK4 expression was considerably reduced in the brain tissues of MCAO model animals compared to sham controls (Fig. 1B-D). Similarly, DOCK4 levels decreased significantly in N2a cells, with expression dropping when the reoxygenation phase was extended from 3 to 12 h but increasing after 24 h of reoxygenation (Fig. 1F-H). The 12-h reoxygenation point of the additional OGD/R model was explicitly selected in light of the above results. These findings suggest that the deregulation of miR-181d and DOCK4 may be highly related to IS development and pathogenesis.

### Inhibition of miR-181d alleviates OGD/R-induced cell injury

**Fig. 2:**
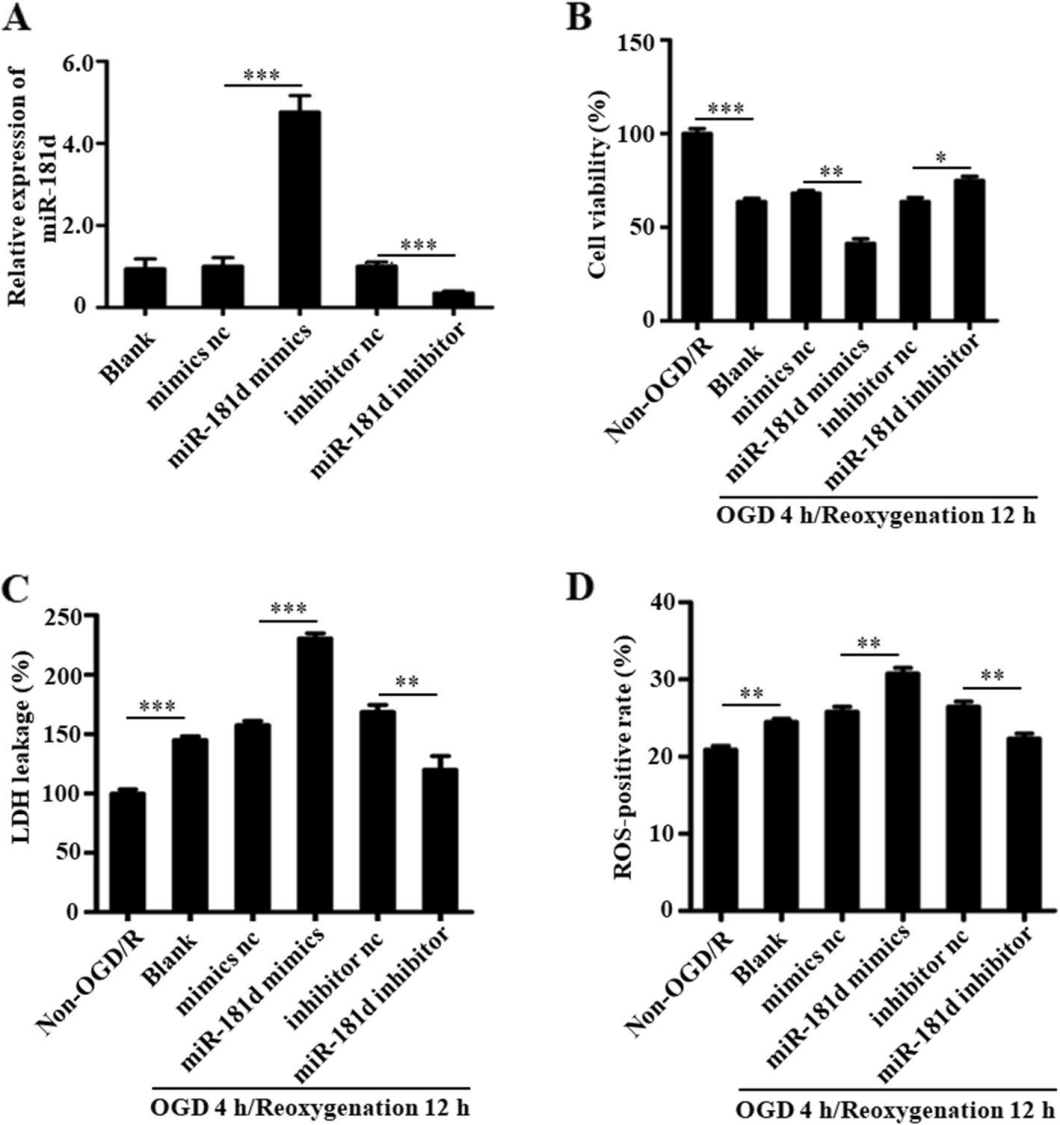
Inhibition of miR-181d alleviates OGD/R-induced cell injury. **A** Relative expression of miR-181d in N2a cells transfected with miR-181d mimics, inhibitor or respective controls was measured by qRT–PCR. **B** MTT assay showed that cell viability was significantly decreased in N2a cells transfected with miR-181d mimics and increased in N2a cells transfected with miR-181d inhibitor (antagomir) after OGD/R treatment. **C** LDH assay showed that overexpression of miR-181d substantially aggravated cell death, while inhibition of miR-181d suppressed cell death induced by 4 h OGD/12 h reoxygenation. **D** After transfection with appropriate mimics, inhibitor, and respective controls for 24 h as appropriate followed by a 4 h OGD/12 h reoxygenation exposure, flow cytometry was utilized to measure ROS production in N2a cells. Overexpression of miR-181d substantially facilitated ROS production, while inhibition of miR-181d reduced ROS production in N2a cells after OGD/R treatment (*P < 0.05, **P < 0.01, ***P < 0.001)

To ascertain the precise pathogenic role of miR-181d in OGD/R-induced cell injury in N2a cells, this study used miR-181d inhibitor or miR-181d mimic transfection (Fig. 2A) and examined the effects on OGD/R-induced cell damage. The MTT experiment demonstrated that silencing miR-181d significantly increased the viability of N2a cells when treated with OGD/R (Fig. 2B). The LDH assay showed that silencing miR-181d significantly reduced OGD/R-induced cell damage (Fig. 2C). ROS levels were dramatically enhanced by OGD/R therapy, whereas miR-181d suppression significantly decreased ROS production. When miR-181d was overexpressed, ROS production was enhanced in response to OGD/R exposure (Fig. 2D). Based on these findings, it is proposed that miR-181d suppression reduces oxidative injury to cells during the OGD/R injury response.

### miR-181d inhibition attenuates OGD/R-induced cell apoptosis

**Fig. 3:**
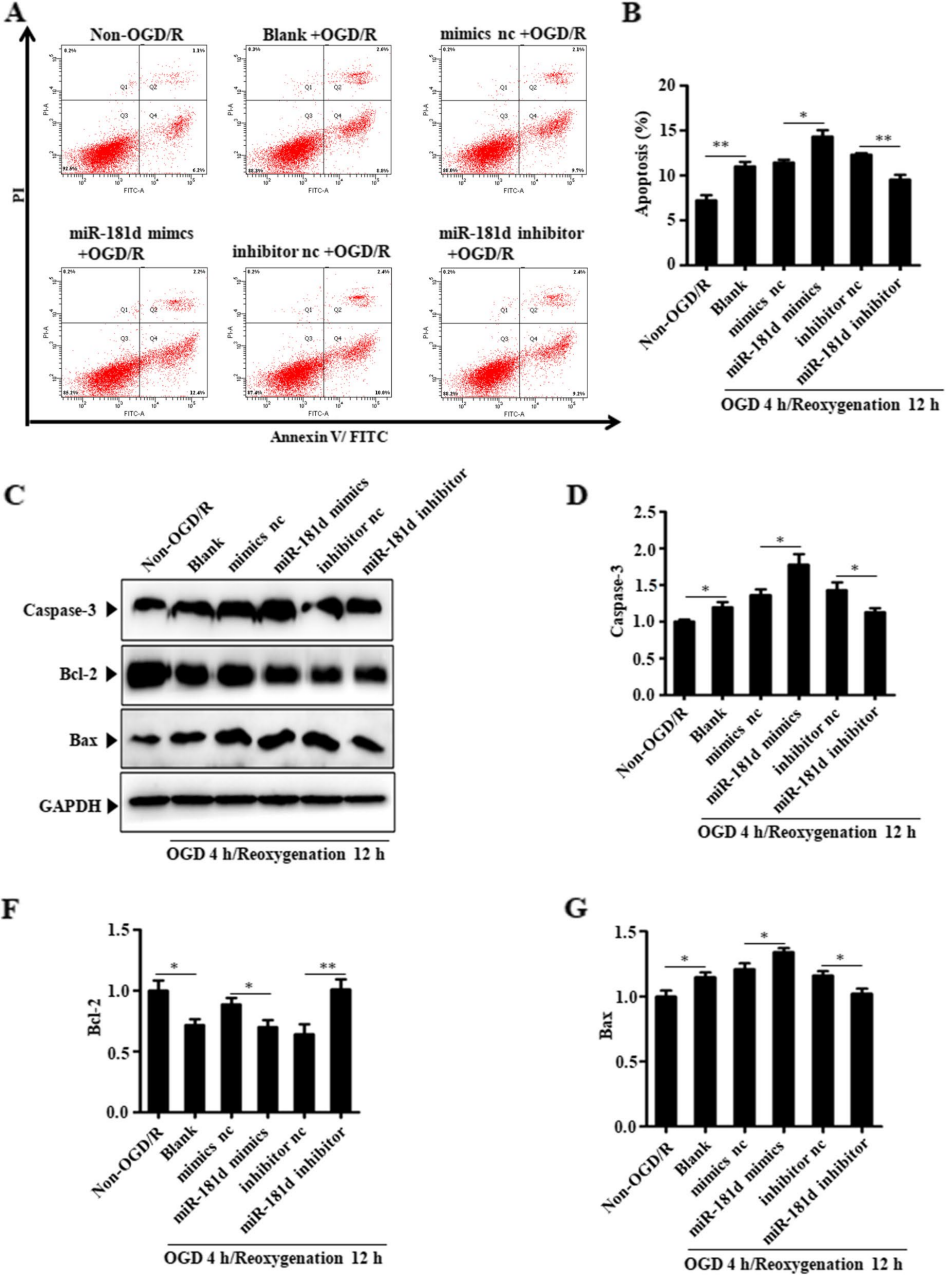
Inhibition of miR-181d leads to reduced N2a cell apoptosis in response to OGD/R conditions. **A** After transfection with appropriate mimics, inhibitor, and respective controls for 24 h as appropriate followed by a 4 h OGD/12 h reoxygenation exposure, cell apoptosis was determined by Annexin V-FITC/PI staining in N2a cells. **B** The apoptotic cell rate is also shown in each group. **C** Western blotting was used to assay the expression of apoptosis-related caspase3, Bcl-2 and Bax in N2a cells prior to transfection with appropriate mimics, inhibitor, and respective controls for 24 h as appropriate prior to use in a 4 h OGD/12 h reoxygenation model. **D**-**F** Relative expression levels of caspase3, Bcl-2 and Bax were quantified and normalized (*P < 0.05, **P < 0.01)

Additionally, the assessment of how miR-181d expression affects cellular apoptosis using Annexin V-FITC/PI staining revealed that cellular apoptosis was suggestively elevated following OGD/R therapy. Silencing or overexpression of miR-181d significantly decreased or enhanced the frequency of OGD/R-induced apoptosis, respectively (Fig. 3A&B). Additionally, western blotting was employed to examine the expression of apoptosis-linked proteins, including cleaved caspase-3, Bax, and Bcl-2. It was discovered that miR-181d inhibition decreased cleaved caspase-3 and Bax levels and improved Bcl2 expression, whereas miR-181d overexpression resulted in the opposite phenotype (Fig. 3C-G).

### miR-181d directly targets DOCK4

**Fig. 4:**
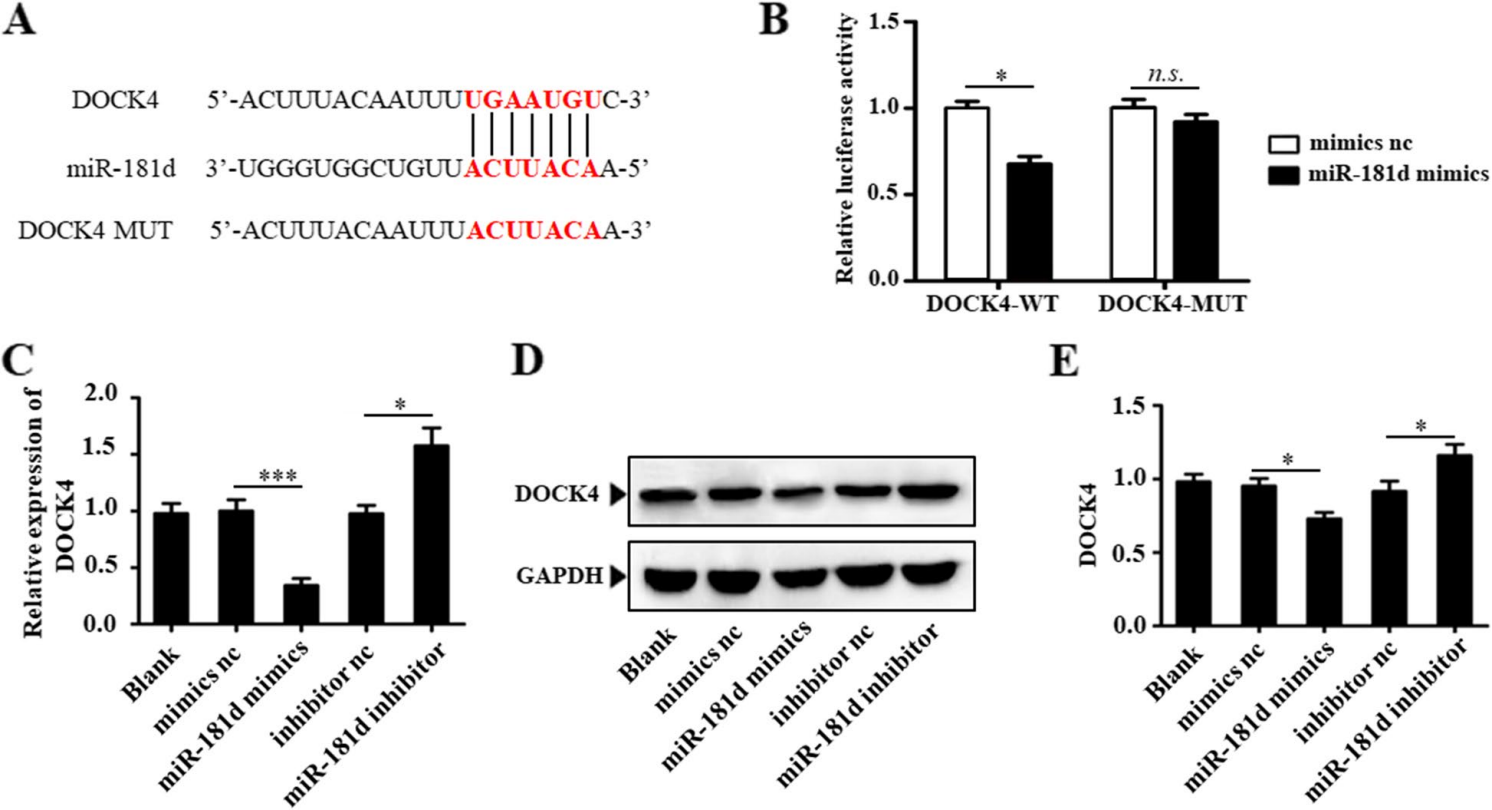
miR-181d directly targets DOCK4. **A** Alignment of miR-181d with WT DOCK4-3′UTR and MUT-DOCK4-3′UTR showing complementary pairing. **B** Dual luciferase assay was performed in N2a cells by cotransfection with the WT DOCK4-3′UTR or MUT-DOCK4-3′UTR and with miR-181d mimics or corresponding controls. qRT–PCR (**C**) and western blotting (**D**) were used to detect the expression of DOCK4 in N2a cells transfected with miR-181d mimics, inhibitor or their respective controls. **E** Relative protein expression of DOCK4 was quantified and normalized (n.s., not significant, *P < 0.05, ***P < 0.001)

DOCK4 is a likely target gene for miR-181d, according to the bioinformatic analysis (Fig. 4A). The luciferase reporter experiment revealed that miR-181d regulates DOCK4 in the event of CI/RI. The findings show that miR-181d overexpression caused a substantial decrease in luciferase activity for constructs comprising the WT DOCK4 3′-UTR. At the same time, this was contentious when the miR-181d-DOCK4 pairing site was altered in these 3′-UTR regions (Fig. 4B). Meanwhile, transfection with miR-181d mimics in N2a cells was linked with decreased DOCK4 protein levels, whereas the opposing phenotype was detected when miR-181d was inhibited (Fig. 4C-E). According to the study, as mentioned earlier, miR-181d can bind to the DOCK4 3′-UTR and control mRNA production.

### miR-181d targets DOCK4 to aggravate OGD/R-induced cell injury

**Fig. 5:**
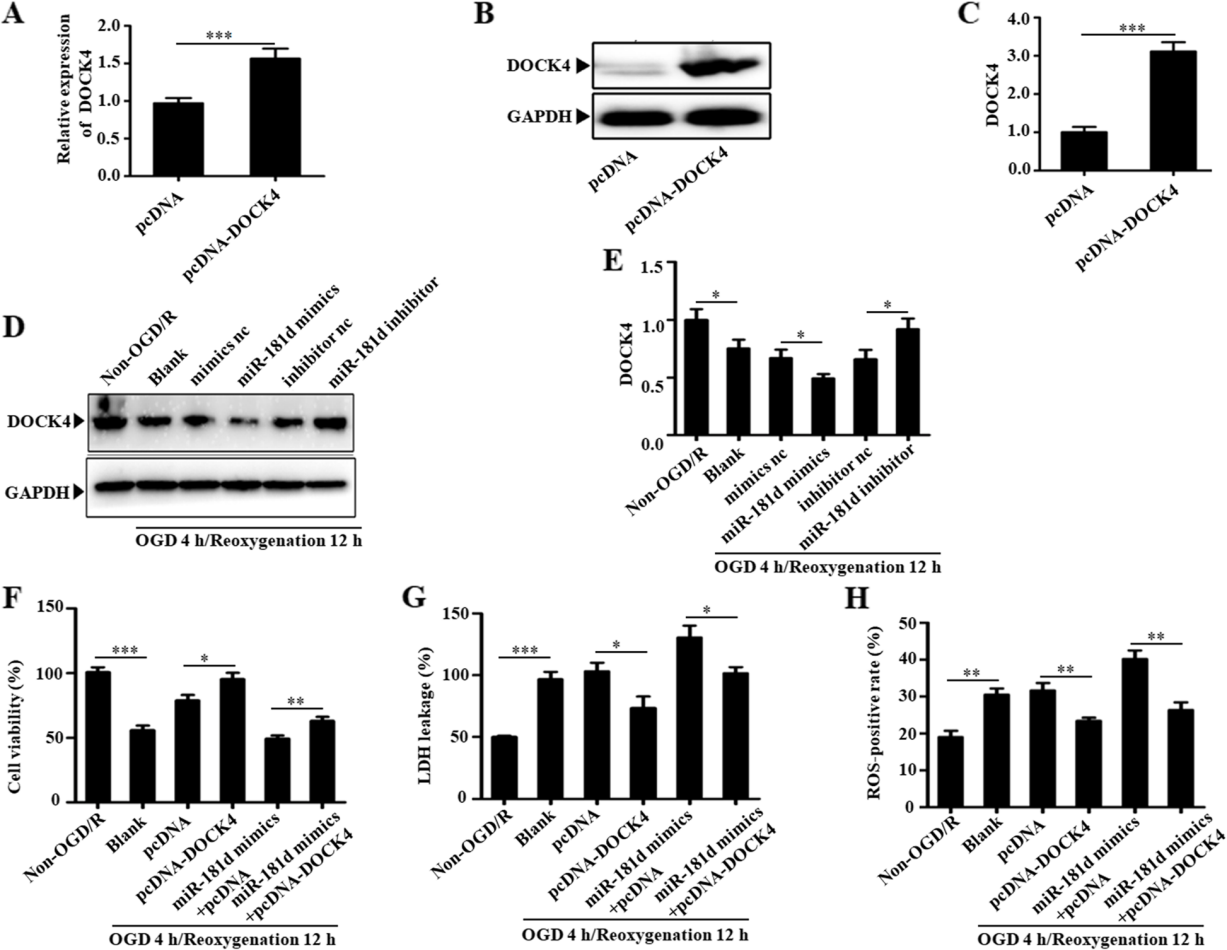
miR-181d targets DOCK4 to aggravate cell injury in response to OGD/R. Constructs harboring full-length DOCK4 (pcDNA-DOCK4) were prepared. pcDNA-DOCK4 efficiently overexpressed DOCK4 at both the mRNA level (**A**) and protein level (**B** and **C**). The expression of DOCK4 in N2a cells transfected with miR-181d mimics, inhibitor or respective controls after OGD/R treatment (**D** and **E**); **F** MTT assay showed that pcDNA-DOCK4 improved cell viability decreased by miR-181d overexpression; **G** LDH assay indicated that pcDNA-DOCK4 alleviated cell injury caused by miR-181d overexpression; **H** pcDNA-DOCK4 reduced the production of ROS caused by miR-181d overexpression (*P < 0.05, **P < 0.01, ***P < 0.001)

The next step was to determine whether the ability of miR-181d to increase cell I/R damage was related to its inhibition of DOCK4 expression. First, this work developed frameworks representing the entire length of DOCK4 (pcDNA-DOCK4). When the cells were transfected with pcDNA-DOCK4, a significant increase in DOCK4 expression compared to controls was detected (Fig. 5A-C). Furthermore, miR-181d mimic transfection in N2a cells decreased DOCK4 expression in the framework of OGD/R therapy, whereas miR-181d inhibition caused the opposite phenotype (Fig. 5D&E). The potential of miR-181d overexpression to alleviate cell viability, enhance ROS production, and induce cell death was significantly reduced in DOCK4-overexpressing cells (Fig. 5F-H). This demonstrated that DOCK4 overexpression inhibited miR-181d upregulation-induced neuronal damage under CI/RI conditions.

### Overexpression of DOCK4 rescues the ability of miR-181d upregulation to induce cell apoptosis

**Fig. 6:**
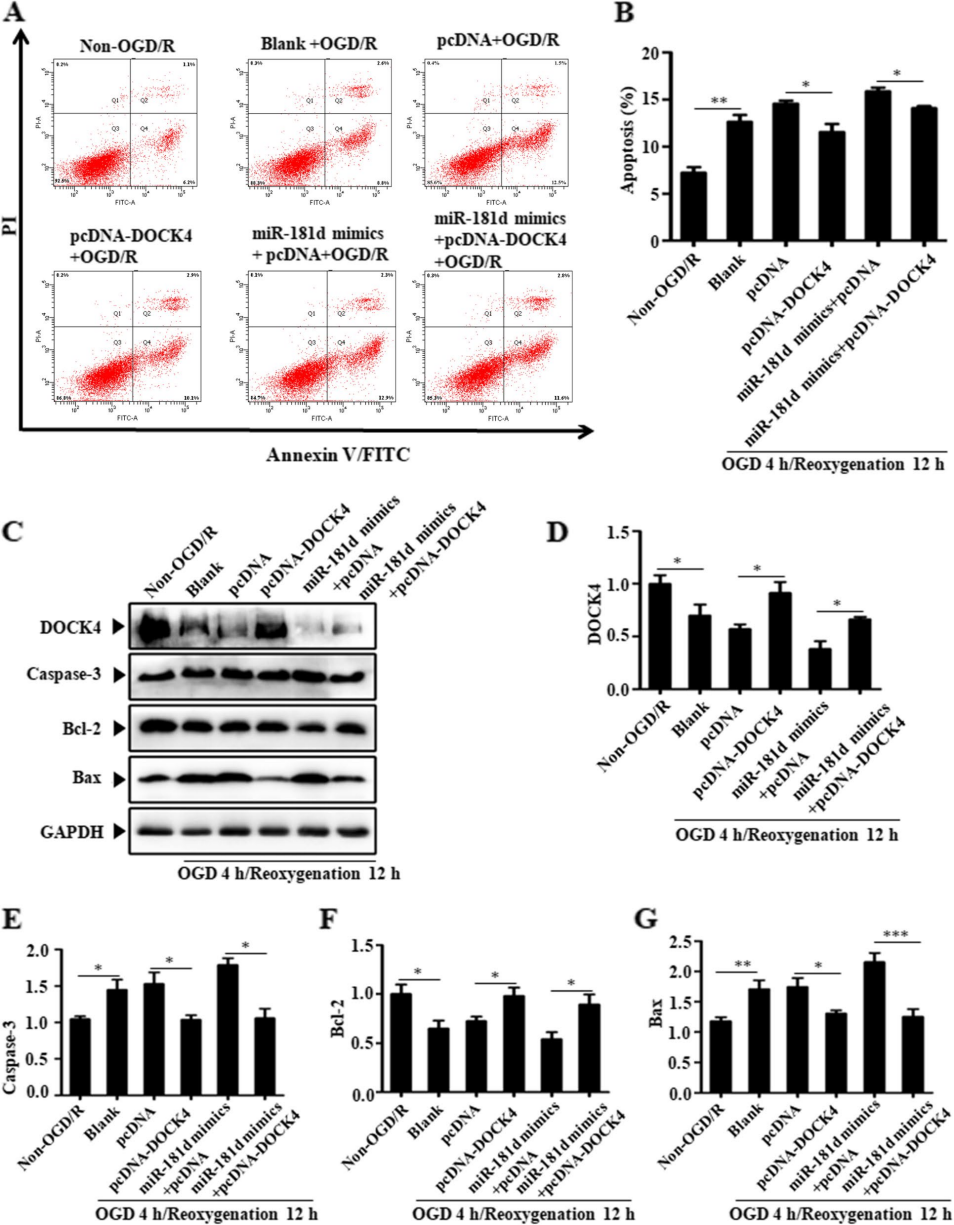
Overexpressing DOCK4 reverses the ability of miR-181d upregulation to induce cell apoptosis. **A** Cell apoptosis was detected by Annexin V-FITC/PI double staining in N2a cells transfected with different groups followed by 4 h OGD/12 h reoxygenation exposure; **B** The apoptotic cell rate is also shown in each group. **C** Western blotting was used to determine the expression of apoptosis-related caspase3, Bcl-2, and Bax as well as DOCK4 in N2a cells transfected with different groups following 4 h OGD/12 h reoxygenation exposure. **D**-**G** Relative protein expression of DOCK4, caspase3, Bcl-2, and Bax was quantified and normalized (*P < 0.05, **P < 0.01, ***P < 0.001)

The results indicated the reliance on miR-181d-mediated control of N2a cell apoptosis caused by OGD/R damage on DOCK4 expression. Compared to control cells, DOCK4 overexpression significantly reduced OGD/R-induced apoptosis. When miR-181d was overexpressed, it accelerated OGD/R-induced apoptosis, whereas DOCK4 overexpression reversed this effect (Fig. 6A&B). Accordingly, Bax and cleaved caspase-3 levels were improved in OGD/R-induced cells, followed by DOCK4 overexpression, while Bcl-2 levels were enhanced. Upregulating miR-181d increased the expression of Bax and cleaved caspase 3 while decreasing Bcl2, while this outcome was reversed in DOCK4-overexpressing cells (Fig. 6C-E). As previously stated, miR-181d induces cellular death in OGD/R-exposed cells by targeting DOCK4.

### DOCK4 variants and the morbidity of IS

The link between DOCK4 mutations and IS has never been investigated. Blood samples were collected from 1086 IS cases and 1045 healthy controls. Table S1 (Supplementary Table 1) provides basic information on stroke patients and healthy controls. No significant differences were observed regarding age, total cholesterol, serum uric acid, or low-density lipoprotein (LDL) between the two groups. However, there were variations in sex, smoking status, diabetes, and high blood pressure values between the IS and the controls. The IS group had higher homocysteine (HCY) and triglyceride levels but lower high-density lipoprotein (HDL) cholesterol levels than the control subjects.

The genotype distribution and allelic frequency of the DOCK4 variations are shown in Table 1. The tested variations in both groups did not vary from Hardy Weinberg equilibrium (*P* > 0.05). However, a genotypically linked study of IS cases and controls showed a statistical link between the rs2074130 variations and IS risk (*P* = 0.0056). Significant differences in rs2074130 frequency were observed in a recessive model (CT + TT vs. CC) for IS cases compared with controls (*P* = 0.0012). In contrast, the dominant model (CC + CT vs. TT) revealed a substantial difference in rs2074130 frequency (*P* = 0.012). Compared to the control group, the frequencies of the variant T allele at rs2074130 (*P* = 0.0056) were substantially higher in the IS group. In contrast, no statistical correlation between the rs2217262 variation and IS danger was observed, even when stratified using dominant or recessive models. Furthermore, the T-A haplotype was more frequent in IS cases than in controls (*P* = 0.0099). This haplotype was related to a significantly higher risk of IS after adjusting for sex, age, high blood pressure, smoking, hyperlipidemia, and diabetes mellitus (Table 2).

**Table 1:** Frequencies of DOCK4 genotypes and alleles in IS patients and controls

Genotypes	IS patients *n* = 1086(%)	Controls *n* = 1045(%)	AOR(95%CI)	*P value*	*P value*^a^
**rs2074130**					
CC	871(80.2)	886(84.8)		**0.0013**	**0.0056**
CT	200(18.4)	156(14.9)			
TT	15(1.4)	3(0.3)			
Dominant model CC + CT vs TT	1071(98.6)	1042(99.7)	0.21(0.059-0.71)	**0.0058**	**0.012**
Recessive model CT + TT vs CC	215(19.8)	159(15.2)	1.38(1.10-1.72)	**0.0054**	**0.012**
C allele	1942(89.4)	1928 (92.2)			
T allele	230(10.6)	162(7.8)	1.41(1.14-1.74)	**0.0014**	**0.0056**
**rs2217262**					
AA	955(87.9)	893(85.5)		0.23	0.26
AC	121(11.2)	141(13.5)			
CC	10(0.9)	11(1.0)			
Dominant model AA+AC vs CC	1076(99.1)	1034(98.9)	1.14(0.48-2.71)	0.76	0.76
Recessive model AC + CC vs AA	131(12.1)	152(14.5)	0.81(0.63-1.04)	0.091	0.13
A allele	2031(93.5)	1927(92.2)			
C allele	141(6.5)	163(7.8)	0.82(0.65-1.04)	0.097	0.13

*P* value of difference in genotypes between case group and control group

*P* < 0.05 is indicated in bold font

Adjusted for age, gender, smoking, hypertension, diabetes mellitus and hyperlipidemia

*AOR* Adjusted odds ratio, *CI* Confidence interval, *IS* Ischemic stroke

^a^False discovery rate-adjusted *P* value for multiple hypotheses testing using the Benjamini-Hochberg method

**Table 2:** Haplotype frequencies in cases and controls and their relationship to IS risk

Haplotypes	Case (freq)	Control (freq)	*P* value	*P* value^a^	OR (95% CI)
*DOCK4* (rs2074130, rs2217262)					
C-A	916(42.6)	933(44.6)			
C-C	885(40.7)	811(38.8)	0.12	0.12	1.11(0.97-1.27)
*T*-A	249(10.5)	184(8.7)	**0.0033**	**0.0099**	1.38(1.11-1.70)
T-C	122(5.7)	162(8.4)	0.041	0.062	0.77(0.60-0.99)

Adjusted for age, gender, smoking, hypertension, diabetes mellitus and hyperlipidemia

All those frequency < 0.05 will be ignored in analysis

*P* < 0.05 is indicated in bold font

^a^False discovery rate-adjusted P value for multiple hypotheses testing using the Benjamini-Hochberg method

### Effect of DOCK4 variants on DOCK4 expression

**Fig. 7:**
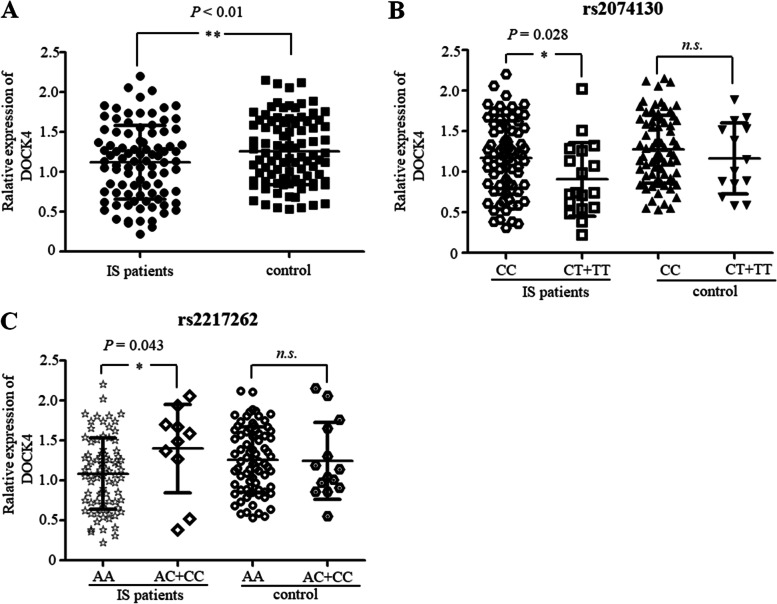
**A** Relative DOCK4 expression in PBMCs from IS patients (*n* = 92) and healthy controls (*n* = 90). Relative GAS5 expression in IS patients and control subjects with rs2074130 CC and CT + TT genotypes (**B**) and rs2217262 AA and AC + CC genotypes (**C**). GAPDH served as the normalization control. Data are expressed as medians with interquartile ranges (*P < 0.05, **P < 0.01). IS: ischemic stroke; n.s.: not significant

DOCK4 expression was measured in the peripheral blood mononuclear cells (PBMCs) of 92 IS cases and 90 healthy controls. The results revealed that mean DOCK4 levels were significantly lower in IS cases than in controls (*P* < 0.01) (Fig. 7A). Furthermore, when the IS patients based on their rs2074130 genotypes were separated, it was found that people with the rs2074130 CT + TT genotype had significantly lower DOCK4 expression than people with the rs2074130 CC genotype (Fig. 7B). Control samples showed no significant differences in DOCK4 expression compared to the rs2074130 CT + TT and CC genotypes (*P* = 0.36). In the case of the rs2217262 variant, no significant difference was found in DOCK4 expression between the mutant allele carriers and the population having the main AA in either patients with IS or control participants (Fig. 7C).

## Discussion

To our knowledge, miR-181d expression and function in the progression of cerebral I/R have not been evaluated. In contrast, miR-181a, miR-181b and miR-181c have been implicated in the pathogenesis of cerebrovascular diseases. In the current investigation, dysregulated miR-181d was discovered in MCAO and reperfusion rats and OGD/R N2a cells. The results indicated that miR-181d expression was increased in both I/R injury models. Significantly, silencing miR-181d boosted cell viability and lowered apoptosis, whereas overexpression of miR-181d exacerbated OGD/R-induced cell damage and increased cellular apoptosis.

Additionally, miR-181d ameliorates cell damage generated by OGD/R by directly targeting DOCK4. Furthermore, the DOCK4 rs2074130 mutation was found to be associated with a high IS risk in a Southern Chinese community. The T-A haplotype was associated with a significant risk of IS susceptibility. Similarly, a substantial reduction in DOCK4 expression was observed in PBMCs from IS patients carrying the rs2074130 CT + TT genotype. The data above indicate that the miR-181d/DOCK4 axis may serve as a novel target for IS therapy.

Numerous reputable studies have established that the miR-181 family plays a key regulatory role in cerebral I/R damage. After stroke, miR-181a antagomir treatment dramatically reduced infarction size, ameliorated neurological impairments, and decreased inflammatory responses, implying therapeutic potential [26]. Inhibition of miR-181b protects MCAO mice against cerebral ischemia [27]. The exosomal miR-181b produced by adipose-derived stem cells promotes angiogenesis of human cerebral microvascular endothelial cells following OGD/R injury by targeting TRPM7 [28]. MiR-181c expression was lower in the plasma and lymphocytes of stroke patients. At the same time, miR-181c suppression increased brain ischemia–reperfusion injury in vivo by increasing microglia and neuron apoptosis via the control of proapoptotic and antiapoptotic responses [29]. However, compared to other miR-181 s, the role of miR-181d in CI/RI has been less addressed. This study is the first to claim that miR-181d is elevated in both in vivo and in vitro models of IS and that miR-181d suppression ameliorates OGD/R-induced cellular injury.

The pathophysiology of neurological disorders is closely associated with the apoptosis of neuronal cells [30], and accumulating evidence has demonstrated that the miR-181 family regulates apoptosis after neuronal cell injury. Inhibiting miR-181a in vivo was linked with increased expression of the apoptotic regulating proteins BCL2 and XIAP, associated with considerably decreased infarction extent and increased neurological deficits [26]. The p38/JNK signaling pathway is inhibited by miR-181b by targeting TLR4 and alleviates autophagy and cellular apoptosis in juvenile epileptic rats [31]. In MCAO mice, miR-181c enhanced infarct volume by suppressing the expression of Bcl-2, increasing the expression of Bax, and activating caspase-3 [29]. MiR-181d suppresses the development of glioma cells and promotes apoptosis and cell cycle arrest via K-RAS and Bcl-2 targeting [32]. Therefore, it is concluded that increased miR-181d levels were linked with increased ROS production and apoptosis in cells treated with OGD/R. Inhibiting the expression of this miRNA showed the opposite effect, indicating that miR-181d increases OGD/R-induced apoptosis under conditions of brain I/R damage.

DOCK4 is a Dock180-related Rho-GEF protein family member. It explicitly activates Rac GTPases, which are required for the cell cycle, cellular connections, cell actin dynamics, vesicular trafficking, cell damage response, axon transport and neurite growth [18, 33]. DOCK4 is expressed in high abundance in rat brains, particularly in hippocampal neurons during late development and adulthood [34]. Ueda et al. showed that DOCK4 was localized in dendritic spines in hippocampal neurons and played a positive regulatory role in dendritic growth and branching [13, 34]. DOCK4 regulates VSMC migration effectively, and its expression is enhanced by PDGF signaling [35]. A recent study discovered that human atherosclerosis has higher levels of DOCK4 and scavenger receptor class-B type-1 (SR-B1), and DOCK4 activates Rac1 by boosting LDL binding to SR-B1, promoting SR-B1 internalization and transport of LDL [19]. Despite these developments, the function of DOCK4 in CIR remains largely unknown. For the first time, miR-181d was validated to target DOCK4 selectively. Furthermore, DOCK4 expression was significantly lowered following OGD/R therapy. Similarly, upregulating DOCK4 expression can reduce oxidative stress and cell death, enhancing full viability in cell injury. Furthermore, DOCK4 overexpression substantially alleviated the increased ROS production and cellular death caused by miR-181d upregulation. This indicates that miR-181d promotes the apoptosis of OGD/R-treated cells by targeting DOCK4.

DOCK4 is present in the AUTS-1 (7q31.1) region, and previous reports have found that it may be susceptible to multiple neuropsychiatric disorders, such as schizophrenia, dyslexia, and ASDs [36, 37]. The DOCK4 rs2217262 allele is linked with autism in both the Caucasian and Han populations in northern China [14, 38]. Shao et al. reported that the DOCK4 rs2074130 variant was related to a high danger of developmental dyslexia [25]. DOCK4 mutation at rs2074130 was associated with a decreased ability to activate downstream Rap1 and Rac1 and failed to promote neurite development and dendritic spine maturation, as well as excitatory synaptic transmission [15]. Although DOCK4 is well known to play an important regulatory role in the pathophysiology of neuropsychiatric disorders, the DOCK4 alleles that are specifically related to IS remain unknown. In the current investigation, DOCK4 rs2074130 variations were strongly associated with a high risk of IS.

Additionally, after adjusting for sex, age, hypertension, smoking, hyperlipidemia, and diabetes mellitus, those bearing the T-A haplotype were 1.38 times more likely to have IS. DOCK4 expression was lower in IS patients with the rs2074130 CT + TT genotype. DOCK4 mutants carrying the rs2074130 mutation could not activate Rac1 and Rap1, impairing their activities in promoting neurite development, synaptic morphogenesis, and transmission [15]. The conclusions of the current study and previous findings suggest that the rs2074130 T allele is linked with decreased DOCK4 expression and/or compromised Rac1 and Rap1 activities, leading to increased susceptibility to IS.

### Comparisons with past studies, as well as what the current work adds to the existing body of knowledge

Previous studies have found that miR-181a, 181b and 181c are involved in the regulation of CI/RI, but the role of miR-181d is not clear. The current study is the first to explore the expression and mechanism of miR-181d in brain I/R injury. In addition, the newly discovered miR-181d/DOCK4 axis may represent a unique therapeutic target for IS. The association between DOCK4 polymorphisms and IS has not been determined so far. Our current study shows that the DOCK4 rs2074130 polymorphism may alter susceptibility to IS by controlling DOCK4 expression, which has substantial implications for the prevention and individualized therapy of IS.

### Study strengths and limitations

This study has several strengths. The miR-181d/DOCK4 pathway in cerebral I/R injury has never been studied before, and this study shows that DOCK4 expression compensation can partially reverse the effect of miR-181d. This suggests that miR-181d regulates cerebral I/R injury by negatively targeting DOCK4. This study also investigated the linkage of DOCK4 variants and IS risk. Haplotype analysis examined the association of DOCK4 haplotypes with susceptibility to IS in over 1000 cases and control subjects. Multiple hypothesis tests were also conducted using the Benjamini–Hochberg method to lower the false discovery rate.

Several limitations need to be considered. First, the expression of Rac1 or Rap1, which are downstream effectors of DOCK4, was not determined, implying that the effect of DOCK4 and its variations on Rac1 or Rap1 activation in the IS context was unknown. Second, the mechanism by which the miR-181d/DOCK4 axis promotes I/R damage in the brain should be validated in vivo. Third, the Han people of southern China make up the only focus of our study population. The current findings must be confirmed in multicentric populations of various ethnic groupings because genetic predisposition is intimately tied to ethnic background. Additionally, further research is needed to determine the mechanism through which DOCK4 SNPs contribute to IS pathogenesis.

## Conclusion

In conclusion, the current work is the first to reveal the upregulation of miR-181d expression in various brain I/R damage models both in vivo and in vitro. Furthermore, inhibiting miR-181d enhances neuronal endurance following OGD/R by targeting DOCK4 directly. This suggests that a therapeutic target for IS treatment could be the miR-181d/DOCK4 axis. The Han Chinese population is at high risk for the DOCK4 rs2074130 T allele. As a result, DOCK4 genotyping may identify people at risk for IS and establish individualized prevention and treatment programs.

## Supplementary Information

**Additional file 1: Table S1.** Characteristics of IS cases and controls.

## Data Availability

The datasets supporting the conclusions of this article are included within the article.

## Abbreviations

miR−/miRNA: microRNA
I/R: Ischemia/reperfusion
OGD/R: Oxygen-glucose deficiency/reoxygenation
IS: Ischemic stroke
DOCK4: Dedicator of cytokinesis 4
VSMC: Vascular smooth muscle cell
LDL: Low density lipoprotein
tPA: Tissue plasminogen activator
MCAO: Middle cerebral artery occlusion
SD: Sprague Dawley
DMEM: Dulbecco’s Modified Eagle’s Medium
RT–qPCR: Real-time quantitative polymerase chain reaction
LDH: Lactate dehydrogenase
DCFH-DA: Dichlorofluoresceindiacetate
CT: Computed tomography
MRI: Magnetic resonance imaging
SD: Standard deviation
HWE: Hardy-Weinberg equilibrium
OR: Odds ratios
CI: Confidence interval
