# Supplementary material for: Direct targeting of DOCK4 by miRNA-181d in oxygen-glucose deprivation/reoxygenation-mediated neuronal injury
Source: Lipids Health Dis. 2023 Mar 7;22:34. doi: 10.1186/s12944-023-01794-3 (PMC9990210; doi:10.1186/s12944-023-01794-3)
Supplement: Supplementary file 1 — Additional file 1: Table S1. Characteristics of IS cases and controls. [file 12944_2023_1794_MOESM1_ESM.docx]

Table S1. Characteristics of IS cases and controls.

| Variables | IS patients (n=1086) | Controls  (n=1045) | *P value* |
| --- | --- | --- | --- |
| Mean age (years) | 65.3±9.3 | 65.5±8.2 | 0.60 |
| Male/female | 738/348 | 511/534 | **< 0.001** |
| Smokers, n (%) | 304(28.0) | 125(12.0) | **< 0.001** |
| Hypertension, n (%) | 828(76.2) | 341(32.6) | **< 0.001** |
| Diabetes, n (%) | 366(33.7) | 115(11.0) | **< 0.001** |
| Uric acid (mmol/L) | 316.5±91.7 | 313.6±89.8 | 0.46 |
| Total cholesterol (mmol/L) | 5.09±1.03 | 5.11±1.05 | 0.66 |
| Triglycerides (mmol/L) | 1.53±1.06 | 1.42±0.95 | **0.012** |
| HDL-cholesterol (mmol/L) | 1.36±0.41 | 1.46±0.53 | **< 0.001** |
| LDL-cholesterol (mmol/L) | 3.08±1.03 | 3.03±0.97 | 0.25 |
| HCY (mmol/L) | 10.82±6.12 | 9.82±3.45 | **< 0.001** |

HCY: Homocysteine; HDL: high-density lipoprotein; IS: ischemic stroke; LDL: low-density lipoprotein;

Continuous data are presented as the mean ± standard deviation, median (range) or n (%)

P < 0.05 is indicated in bold font.
